# Immunohistochemical localization of hepatopancreatic phospholipase A_2 _*in Hexaplex Trunculus *digestive cells

**DOI:** 10.1186/1476-511X-10-91

**Published:** 2011-06-01

**Authors:** Zied Zarai, Nicholas Boulais, Aida Karray, Laurent Misery, Sofiane Bezzine, Tarek Rebai, Youssef Gargouri, Hafedh Mejdoub

**Affiliations:** 1 Laboratoire de biochimie et de génie enzymatique des lipases, ENIS BPW 1173 Université de Sfax-Tunisia; 2 Laboratoire de Neurobiologie Cutanée, CHU Morvan, Université de Brest, 29609 BREST cedex France; 3 Laboratoire d'Histoembryologie, FMS, BPW 3029 Université de Sfax, Tunisia

## Abstract

**Background:**

Mammalian sPLA_2_-IB localization cell are well characterized. In contrast, much less is known about aquatic primitive ones. The aquatic world contains a wide variety of living species and, hence represents a great potential for discovering new lipolytic enzymes and the mode of digestion of lipid food.

**Results:**

The marine snail digestive phospholipase A_2 _(mSDPLA_2_) has been previously purified from snail hepatopancreas. The specific polyclonal antibodies were prepared and used for immunohistochimical and immunofluorescence analysis in order to determine the cellular location of mSDPLA_2_. Our results showed essentially that mSDPLA_2 _was detected inside in specific vesicles tentatively named (mSDPLA_2_+) granules of the digestive cells. No immunolabelling was observed in secretory zymogene-like cells. This immunocytolocalization indicates that lipid digestion in the snail might occur in specific granules inside the digestive cells.

**Conclusion:**

The cellular location of mSDPLA_2 _suggests that intracellular phospholipids digestion, like other food components digestion of snail diet, occurs in these digestive cells. The hepatopancreas of *H. trunculus *has been pointed out as the main region for digestion, absorption and storage of lipids.

## Introduction

The Muricidae family of snails includes about 1,000 species, which represent a diverse and important component of marine communities [1]. The banded murex, *Hexaplex trunculus *(Linnaeus, 1758), is found in the mediterranean sea and adjacent atlantic ocean from the Portuguese coast, southward to Morocco and to the Madeira and Canary islands [2] and [3]. This specie is a commercially important marine snail in the mediterranean coasts.

The digestive gland or the hepatopancreas of gastropod molluscs like the marine snail *H. trunculus *is the key organ for metabolism. It is the main source of production of digestive enzymes, and it is involved in absorption of nutrients, food storage and excretion [4]. It combines many functions of the liver, pancreas, intestine and other organs in vertebrates [5]. For this reason, it has captivated scientists for more than 180 years [6] and [7] and it was considered as an excellent model for food digestion and cell secretion [8]. Its primary role is the synthesis and secretion of digestive enzymes, swallowing and final digestion of the ingested food and subsequent uptake of nutrients. Hepatopancreas is also implicated in storage and excretion of inorganic reserves, lipids and carbohydrate metabolites. Moreover, it is the primary metabolic center for the production of materials required for the temporally distinct events of molt and vitellogenesis [9].

Furthermore, the ultrastructure and functional aspects of several aquatic invertebrate hepatopancreases were studied. Crustaceans and mollusc hepatopancreases, similar to that of the marine snail, show mainly two specialized cell types in the digestive diverticula [10] and [11]. Secretory zymogene-like cells which are responsible for extracellular digestion, while digestive cells are involved in absorption, intracellular digestion and nutrients transport. Unfortunately, the digestive process is not thoroughly investigated in invertebrate and the digestive processes remain unclear.

We previously purified a new marin snail digestive phospholipase A_2 _(mSDPLA_2_) from the hepatopancreas of *H. trunculus *[12]. This mSDPLA_2 _of 30 kDa, which contrasts with common 14 kDa-digestive PLA_2_, is of interest as it exhibites hemolytic properties and could be used as model to study digestive and cytotoxicity mechanisms. Zarai et al [12] have shown that the potential mSPLA2 activity was measured, *in vitro*, in presence of bile salts like NaTDC or NaDC. This result confirms that mSPLA_2 _presents a high interaction power which allows it to bind to its substrate even in presence of bile salts.

Many studies have described the morphology as well as the digestive system of snails. The hepatopancreas and the salivary glands of the snails are the main sources of digestives enzymes. To our knowledge, the presence of bile salts in the molluscs digestive glands has never been described. As recently shown by Amara et al (2010) [13] no bile salts are detected in the land snail digestive gland. One can say that, *in vivo*, the digestive system of sail contains probably some molecules (bile salts-like) which allow the snail phospholipase to hydrolyse efficiently its substrate.

To investigate the digestive function of mSDPLA_2 _in invertebrate, and to elucidate its digestive mechanism in hepatopancreas, we performed immunohistochemistry and immunofluorescence analysis using rabbit anti-mSDPLA_2 _whole serum. Our current studies demonstrate that marine snail mSDPLA_2 _has both intracellularly and extracellularly food digestion role. The digestive processes by mSDPLA_2 _were accurately followed by the use of the anti-mSDPLA_2 _polyclonal antibodies. A digestive model in hepatopancreas is therefore formulated.

## Results

### General digestive gland description

The hepatopancreas of molluscs is a large digestive gland formed by a vast number of blind ending tubules; the digestive diverticula. This organ is involved in several functions including the extracellular and intracellular food digestion, lipids, glycogen and minerals storage. It is also the main site of nutrient absorption and plays a major role in detoxification [14-17].

**Figure 1:**
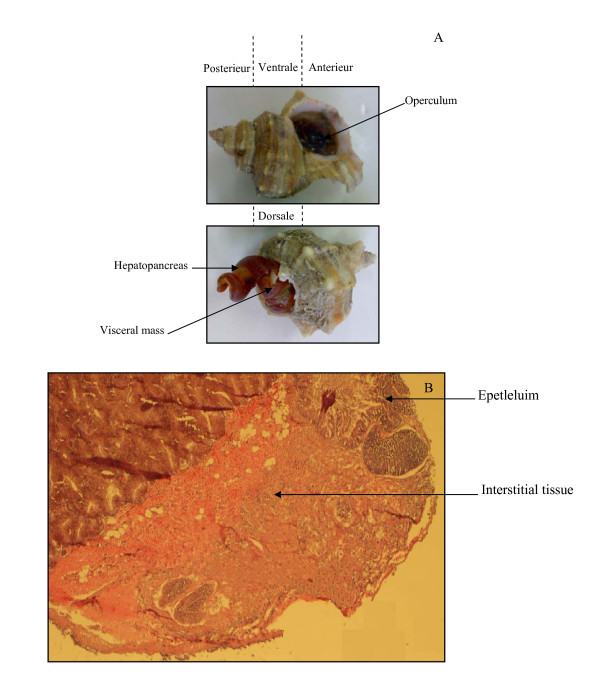
***Hexaplex trunculus***. (A), *Hexaplex trunculus*, the shell has been removed in the posterieur side but the mantle is intact, marine snail sagittale section showing the hepatopancreas in the posterieur side. (B), Hexaplex trunculus hepatopancreas showing the epithelium of the digestive diverticula displaying the small lumen and the interstitial tissue. Sections were stained with eosine and hematoxyline.

*H. trunculus*, like other molluscs, has an acini digestive glands. The lumen of acinous gland communicates by ducts with stomach lumen. The epithelium lining the tubules of the glands consists chiefly of tall columnar cells; their apical surface is typically covered by microvilli (Figure 1).

The digestive diverticula consist of an epithelium with a single layer of cells, separated from the surrounding connective tissue and muscle cells by a basal lamina. In several molluscs these epitheliums consist of the digestive and basophilic cells [18-21]. However, in gastropods other cell types have been reported in addition to these two cell types [22-24]. Digestive cells are the most abundant cell type in the digestive diverticula of *H. trunculus*.

Molluscs digestive cells are mainly characterized by the presence of a large number of heterolysosomes, in which food digestion is completed. These columnar shaped cells are the most abundant in the hepatopancreas and their apical surface is covered by microvilli. Lipid droplets and glycogen granules are usually present in the cytoplasm of digestive cells. The basophilic cells, also called secretory or crypt cells, are typical the main protein secreting cells. These pyramidal-shaped cells contain large amounts of rough endoplasmic reticulum, a well-developed Golgi complex and accumulate secretion granules. They seem to be responsible for the secretion of digestive enzymes, which undertake the extracellular food digestion [18-20,25,23]. In mollusc hepatopancreas, we suppose that duct cells can be involved in lipid storage and nutrient absorption [26]. Moreover, some results suggest that these cells may play a role in digestion [25].

### Immunodetection of mSDPLA_2 _in the crude hepatopancreas homogenate

**Figure 2:**
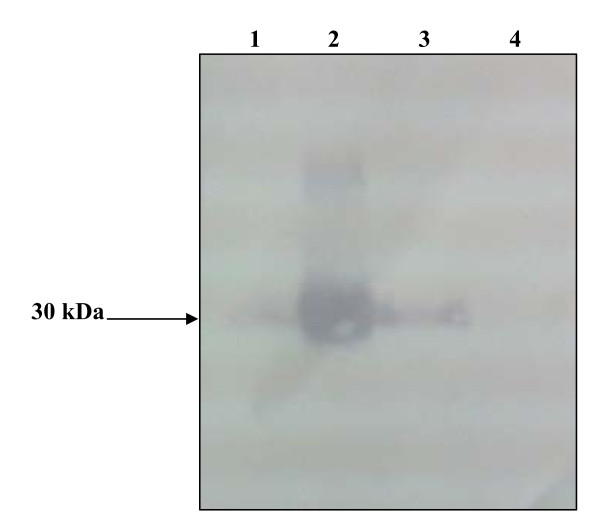
**Immunoblot**. Immunoblot analysis of 5 μg mSDPLA_2 _(lane 1); 50 μg mSDPLA_2 _(lane 2); 15 μg mSDPLA_2 _(lane 3); PPLA_2 _(lane 4). Using mSDPLA_2 _serum at 1: 1000 dilution.

Supernatant of the marine snail hepatopancreas homogenate containing 100 μg of total proteins was subjected to SDS-PAGE analysis followed by immunoblotting. Anti-mSDPLA_2 _polyclonal antibodies were found to react with a single band of 30 kDa corresponding to the mSDPLA_2 _present in the crude extract. No other protein bands were recognized by anti-mSDPLA2, suggesting a good specificity of these mSDPLA_2 _antibodies (Figure 2). Based on the specificity towards mSDPLA_2_, polyclonal antibodies were used for immunocytolocalization of mSDPLA_2 _in the hepatopancreas sections.

### Immunocytolocalization of mSDPLA_2_

**Figure 3:**
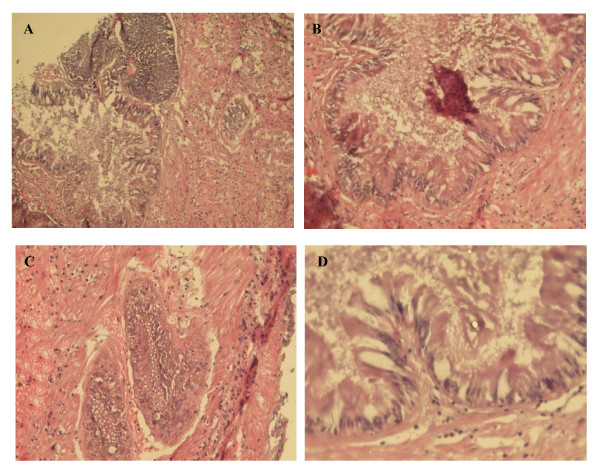
***Hexaplex trunculus *Cryostat tissue sections (× 400) through digestive diverticula**. Frozen 4 μm sections from *H. trunculus *hepatopancreas were used for control experiments (without pAbs anti-mSDPLA_2_) and stained with eosine and hematoxyline for genral morphology. No labeling was observed without pAbs anti-mSDPLA_2_. (A and B) Overall view of a control section of the digestive diverticula at low magnification (100×). (C and D) Enlarged view of the digestive diverticula sectioned longitudinally (400×).

**Figure 4:**
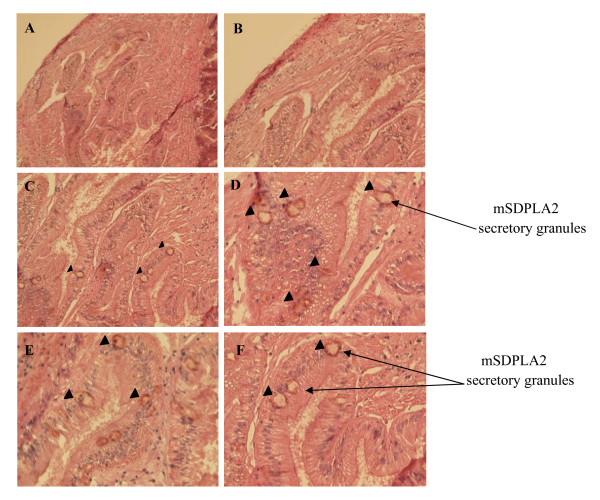
**Immunalabelling of the digestive gland**. (A and B) Overall view of a sections of the digestive diverticula after incubation with the solution containing pAbs anti-mSDPLA_2 _(1:100) and then hybridized with appropriate secondary biotinylated- antibodies (1:200) counter-stained with eosine and hematoxylin. (C and D) The sectioon of digestive diverticula showing the occurrence of mSDPLA_2 _in the lumen of digestive cells, mSDPLA_2 _localize in the around cells. (E and F) enlarged view of the digestive cells (400×).

**Figure 5:**
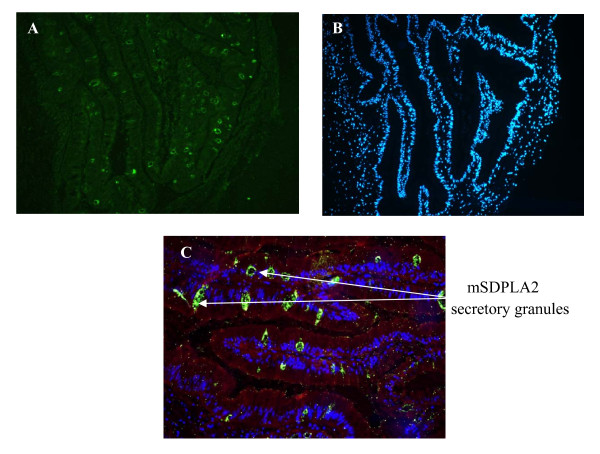
**Immunofluorescence localization of mSDPLA_2 _in a 4-μm cryostat section**. (A) One section was first stained with Di Aminido Phenyl Indol (DAPI) for general morphology. (B) Followed by pAbs anti-mSDPLA_2 _(1:100) revealed by fluorochrome (chromeo) conjugated with the secondary antibody in PBS (1:200). (C) represents the superposition of two images (A and B), showing labelling of digestive cells intracellular granules.

Sections of hepatopancreas were immunostained against mSDPLA_2 _as described above (Figure 4). Only digestive cells displayed a positive labelling for the presence of mSDPLA_2_. Conversely, secretory zymogene-like cells were not immunostained. In the control experiments without anti-mSDPLA_2 _antibodies, no labeling was observed (Figure 3). Interestingly, we noticed that only few intracellular granules of the digestive cells were immunoreactive. These granules containing mSDPLA_2_, tentatively named mSDPLA_2_+ granules, which were irregular in shape and did not have a specific location in the digestive diverticula (Figure 4 and 5). To further confirm the presence of mSDPLA_2 _and the specificity of the mSDPLA_2 _polyclonal antibodies, we performed a laser capture microdissection targeting stained cells of hepatopancreas tissue sections. Selected cells were dissociated and protein extracts were analysed by western-blot. Western blotting revealed a strong broad band at around 30 kDa, corresponding to the mSDPLA_2 _(Data not showed).

## Discussion

The ultrastructure of the hepatopancreas was described in some Opisthobranchs [27-30,24], but such studies were never carried out on Neogastropoda, which includes the genus *Hexaplex*. To enlarge our knowledge about these marine molluscs, the structure and function of the digestive gland of the gastropod mollusc, *H. trunculus*, was investigated through electron microscopy analyses and immunohistochemistry.

The digestive gland is composed of two main cell types, the "digestive" cells and the "secretory" cells. The digestive cells appear to be concerned with the absorption and digestion of nutrients, while the secretory cells produce digestive enzymes and calcareous concretions. Undifferentiated cells are scattered between these two cell types.

In previous studies, we have purified and biochemically characterized an original marine snail digestive phospholipase A_2 _from the hepatopancreas [12]. Here, we report the ultrastructural study and some cytological aspects of the digestive cells. We performed immunocytochemical and immunofluorescence analysis to specify the tissular and subcellular location of this digestive enzyme. We showed that mSDPLA_2 _is located in the digestive cells while no labeling was observed in the secretory zymogene-like cells.

**Figure 6:**
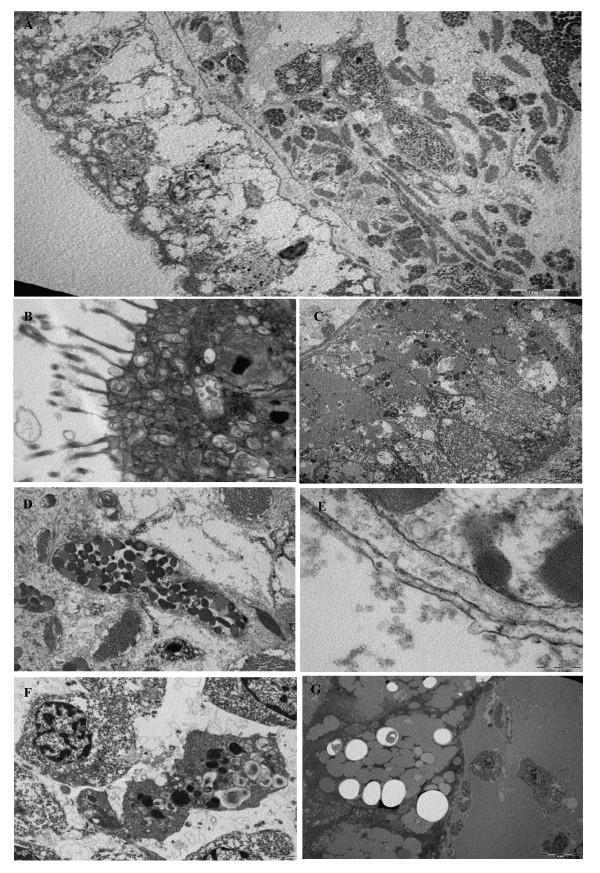
**Electron micrographs of the digestive cells**. (A, B,) the apical region of the cell contains a bruch border of microvilli and numerous clumps of α-glucogen granules. (C) digestive adjacent cells. Note some microtubules (arrow) and a ciluim root (arrowhead). (D) The cytoplasm of digestive cells contain granules similar to hemozoin, vesicles with protein content and lipid droplets, indicating the possible function in digestion, absorption, and storage of lipids. (E) The pinocytotic vesicles fuse together to form heterogeneous phagosomes known as heterophagosomes. (F) A large residual body is about to extruded into lumen at the end of the process. (G) The vacuola become bigger and distributed through the whole cell.

From the granular labeling observed in the digestive cells, mSDPLA_2 _appears as an intracellular enzyme involved in the intracellular food digestion process as described for other invertebrates [31]. In mammals, classical digestive enzymes as pancreatic enzymes were detected in intracellular zymogene granules in pancreatic acinar cells. However they act in the lumen of the gastrointestinal tract which supposes a secretory process from acinar cell [32]. The presence of mSDPLA_2 _in the digestive cells is not a definitive argument for involving it in phospholipid digestion mechanism located inside these cells. Invertebrate digestive cells can take up partially digested food by endocytosis through the microvilli. It was shown that, using this process, digestive cells of a mollusc *Sepia officinalis *absorb 80% of a radiolabeled food source [33]. According to Boucaud-Camou and Yim [34], the pinocytotic vesicles fuse together to form heterogeneous phagosomes known as heterophagosomes (Figure 6E). When they combine with primary lysosomes containing the intracellular digestive enzymes, they form secondary lysosomes or heterolysosomes, where the intracellular digestion takes place. We noted that the mSDPLA_2 _immunoreactive (mSDPLA_2_+) granules belonging to the digestive cells were irregular in shape and size. Small mSDPLA_2_+ granules of about 2 μm might be primary lysosomes or lysosomes-like vesicles. However, larger mSDPLA_2_+ granules of about 7 μm were most likely secondary lysosomes or heterolysosomes. That can result from the fusion of lysosome-like vesicles and phagosomes (Figure 4). The larger dense granules lacking mSDPLA_2 _take a large part of digestive cells are thought to be phagosomes. Furthermore, similar vesicles were previously observed in digestive cells of many invertebrates [35,36]. The number and size of such vesicles can be related to the digestion stage.

The secretory zymogene-like cells must be responsible for secretion of digestive enzymes. They undertake an initial and rapid extracellular food hydrolysis in the diverticula lumen. Then, the partially digested products are likely to be absorbed by pinocytosis (Figure 6E) and stored in the digestive cell where they will be slowly hydrolyzed by intracellular enzymes for energy generation.

The anatomy and the histology of the molluscs digestive system have been previously studied. However, the knowledge of the ultrastructure and the physiology of the *H. trunculus *digestive system remain unknown (poor). In this histologic study, the ultrastructure of digestive cells of *H. trunculus *was investigated by electron microscopy transmission and immunoflourescence.

In semi-thin sections of *H. trunculus *hepatopancreas, many digestive were observed in the epithelium of the digestive diverticula. Digestive cells exhibited numerous heterolysosomes sometime fused together or with phagosomes (Figure 6A and 6B). The apical surface of digestive cells was covered with microvilli, almost could reach 10 μm in length (Figure 6C). We describe a second cell types for which a single large vacuole occupying almost entirely the cell is observed, while the cytoplasm is reduced to a very thin peripheral layer (Figure 6C).

In digestive cells, the apical zone contained many endocytic vesicles with electron dense materials, in addition to vacuoles with a clear background, usually containing low amounts of dense material. A large part of the cytoplasm was filled with heterolysosomes organelles with variable length and contents. This heterogeneity reflects different stages of intracellular digestion.

In molluscs, the extracellular digestion products found in the lumen of digestive diverticula are collected by digestive cells for further breakdown in heterolysosomes. In some species digestive cells can collect relatively large food particles by phagocytosis [37], but in others only dissolved substances are captured by small endocytic vesicles for extracellular digestion [38,39].

For *H. trunculus*, many endocytic vesicles filled with electron dense materials were observed in the apical region of *H. trunculus *digestive cells. Probably, these vesicles contained extracellular digestion products that would be transferred to heterolysosomes of digestive cells, to complete the digestive process. In some digestive cells the number of endocytic vesicles was very high, indicating a very intense endocytic activity (Figure 6E). Conversely, some other cells had, only few vesicles. That suggests the existence of different digestive step at the cellular level, with low endocytic activity. The Golgi stacks with dilated cisternae containing dense substances were detected in digestive cells of *H. trunculus*.

In the hepatopancreas of *H. trunculus*, digestive cells can also be distinguished by size and electron-density of vesicles in their cytoplasm.

Small and electron dense vesicles are associated with secretory functions, whereas large and electron-lucid vesicles are associated with the absorption fonctions. Furthermore, the presence of lipid droplets and glycogen granules in the cytoplasm of digestive cells suggests their involvement in the metabolism of lipids (Figure 6G).

The thorough involvement of the hepatopancrea of *H. trunculus *in secretion, digestion and, metabolism absorption was evidenced by the decondensed aspect of nuclear chromatin, presence of rough endoplasmic reticulum, Golgi complex region, lysosomes, vesicles and cytoplasmic inclusions in the digestive cells. The hepatopancreas of *H. trunculus *has been pointed out as the main region for digestion and Absorption. The cytoplasm of digestive cells contain granules similar to hemozoin, vesicles with protein content and lipid droplets, indicating the possible function in digestion, absorption, and storage of lipids.

## Method and materials

### Animals dissection and tissue collection

The marine snails *H. trunculus *were collected from the sea cost of Sfax, Tunisia (Figure 1). They were kept on ice until use in the laboratory. The carapace and the internal organs were separated and the hepatopancreases were immediately collected and stored at -80°C.

### Preparation of hepatopancreas extracts

Extraction buffer (50 mM Tris-HCl, pH 8.5) was added to the hepatopancreas sample in the proportion of 5 ml per 1 g of fresh tissue and stirred for 45 min at room temperature. Then, 2 mM benzamidine was added to the mixture and centrifuged for 30 min at 10,000 × g. The clear supernatant was collected for protein analysis and phospholipase activity measurement.

### Protein concentration and glycosylation analysis

Protein concentration was measured spectrometrically according to the Bradford method [40], using bovine serum album as a standard. The presence of glycan chains in the pure mSDPLA_2 _was checked by anthrone-sulfuric acid method using glucose as a standard [41]**.**

### Production of anti-mSDPLA_2 _polyclonal antibodies

Rabbits were injected subcutaneously and intra-muscularly every 3 weeks with 0.5 mg of purified mSDPLA_2_. The first injection included complete Freund's adjuvant, and the last two injections contained incomplete adjuvant. Anti-mSDPLA_2 _serum was collected and its immunoreactivity was verified.

### SDS-PAGE and immunoblotting Analysis

Analytical polyacrylamide gel electrophoresis of proteins in the presence of sodium dodecyl sulfate was performed as described by Laemmli [42]. The specificity of anti-mSDPLA_2 _polyclonal antibodies was confirmed by protein blotting. Proteins from SDS-PAGE were blotted onto nitrocellulose membranes. Membranes were then rinsed three times with PBS (10 mM phosphate, 150 mM NaCl, pH 7.2), and saturated with 3% powder milk in PBS for 1 h at room temperature. Thereafter, membranes were incubated for 1 h at room temperature with anti-mSDPLA_2 _polyclonal antibodies diluted at 1:1000 in PBS containing 0.05% tween-20 (PBS-T). Afterward, they were washed three times with PBS-T and incubated for 1 h at room temperature with a 1:2000 dilution of alkaline phosphatase-conjugated anti-rabbit immunoglobulin (Sigma). After washing as above, membranes were incubated with 0.3 mg/ml of nitroblue tetrazolium chloride (Sigma), 0.2 mg/ml of 5-bromo-4-chloro-3 indodylphosphate (Sigma) and 0.2 mg/ml of MgCl_2 _to reveal the specific immunoreactivity.

### Histological, immunochemical and immunofluorescence studies

#### Immunohistochemistry

Fragments of tissues, with a size of around 1 mm^3^, were embedded in Optimal Cutting temperature (OCT) and cryopreserved in isopentane chilled on liquid nitrogen. Sections with a thickness of 4 μm were cut. Slides were saturated with 5% of normal goat serum in PBS with 0.05% Triton X-100 for 15 minutes and subsequently hybridized with primary anti-mSDPLA_2 _polyclonal antibodies diluted at 1:200 in Dako Diluent (S3022) for 2 h at 4°C. Slides were rinsed twice and hybridized with (1:200) secondary anti-rabbit biotinylated- antibodies diluted at 1:200 for two hours at room temperature.

#### Immunofluorescence

Tissue sections were fixed in PBS with 4% paraformaldehyde, permeabilized with 0.5% Triton X-100, saturated in 5% normal goat serum in PBS-T and hybridized with anti-mSDPLA_2 _polyclonal antibodies diluted at 1:100 in Dako Diluent (S3022) overnight at 4°C. After two washes, cells were hybridized for 2 h with a chromeo™-488-conjugated secondary antibody from rabbit (Abcam, Cambridge, UK) diluted at 1:200 in PBS. The fluorescence analyses were performed with BX41 Olympus upright microscope and pictures were taken with an Olympus C-5060 digital camera. Control experiments were carried out in absence of anti-mSDPLA_2 _antibody.

#### Electron microscopic study

Fragments of tissues, with a size of about 1 mm^3 ^were fixed in 2.5% glutaraldehyde for 30 minutes, rinsed in 0.1 M cacodylate buffer and post-fixed for one hour with 1% OsO_4_. Tissues were dehydrated in graded concentrations of alcohol, and then in propylene oxide. Thereafter, tissues were embedded in epoxy resin for 24 h at 60°C. Finally, ultrathin sections were cut and observed with a Joel, Jem-1010 Electron Microscope.

## Conclusion

In conclusion, the marine snail digestive phospholipase A_2 _is an enzyme localized in the digestive cells of the snail hepatopancreas. Immunocytolocalization study shows that only one irregular-shaped vesicle type; mSDPLA_2_+ present in the digestive cells of the snail hepatopancreas was found to contain mSDPLA_2_. The enzyme appears to be homogenously distributed in these vesicles. This cellular location suggests that intracellular phospholipids digestion, like other food components digestion of snail diet, occurs in these digestive cells.

## Abbreviations

mSDPLA_2_: marine snail digestive phospholipase A_2_; SDS-PAGE: sodium dodecyl sulfate-polyacrylamide gel electrophoresis; BSA: bovine serum albumin; pAbs: polyclonal antibodies; PBS: phosphate buffer saline; DAPI: Di Aminido Phenyl Indol; OCT: Optimal Cutting temperature.

## Competing interests

The authors declare that they have no competing interests.

## Authors' contributions

ZZ carried out all the studies, analyzed the data and drafted the manuscript. NB, AK and LM helped with the analysis, discussion of the data and correction of the manuscript. SB helped with the correction of the manuscript. TR helped with the discussion of the data. YG and HM participated in the study design and helped to draft the manuscript. All authors have read and approved the final manuscript.
